# Platelet to lymphocyte ratio is a risk factor for failure of non-operative treatment of colonic diverticulitis

**DOI:** 10.1038/s41598-023-31570-3

**Published:** 2023-03-16

**Authors:** Jong Ho Kim, Sang Hyup Han, Jin-Won Lee, Haesung Kim, Jeonghee Han

**Affiliations:** 1 Department of Anesthesiology and Pain Medicine, Chuncheon Sacred Heart Hospital, Hallym University College of Medicine, Chuncheon, 24253 South Korea; 2 Department of Surgery, CHA Bundang Medical Center, CHA University School of Medicine, Seongnam, 13496 South Korea

**Keywords:** Gastrointestinal diseases, Outcomes research, Risk factors

## Abstract

Non-operative treatment is the mainstay of colonic diverticulitis, but some patients require surgery due to non-operative treatment failure. This study aims to identify risk factors for the failure of non-operative treatment of colonic diverticulitis. From January 2011 to December 2020, we retrospectively reviewed 2362 patients with non-operative treatment for first-attack acute diverticulitis. Patients were categorized into non-operative treatment success or failure groups. Clinical characteristics and serum inflammatory markers were analyzed by multivariable logistic regression to determine risk factors for non-operative treatment failure of colonic diverticulitis. Overall, 2.2% (n = 50) of patients underwent delayed surgery within 30 days (median 4.0 [3.0; 8.0]) due to non-operative treatment failure. Multivariable logistic regression identified that platelet to lymphocyte ratio (odds ratio [OR], 4.2; 95% confidence interval [CI], 0.05–0.13; *p* < 0.001), diabetes mellitus (OR, 2.2; 95% CI, 0.01–0.09; *p* = 0.025), left-sided colonic diverticulitis (OR, 4.1; 95% CI, 0.04–0.13; *p* < 0.001), and modified Hinchey classification (OR, 6.2; 95% CI, 0.09–0.17; *p* < 0.001) were risk factors for non-operative treatment failure. Platelet to lymphocyte ratio (PLR) is a potential risk factor for the non-operative treatment failure of acute first-attack colonic diverticulitis. Therefore, patients with higher PLR during non-operative treatment should be monitored with special caution.

## Introduction

Diverticulitis is one of the most common benign diseases and up to 25% of adults with diverticulosis have the potential to develop acute colonic diverticulitis during their lifetime^1,2^. The prevalence of colonic diverticulitis is gradually increasing^3,4^, and several studies have reported increased hospitalizations for diverticulitis^3,5,6^. Recently, with the westernization of dietary habits and the development of diagnostic technology, the incidence of colonic diverticular disease is increasing in Asian countries, including Korea^7^.

Diverticulitis presents a diverse clinical spectrum, from asymptomatic diverticulitis^8–10^ to life-threatening peritonitis^11^. Clinical practice guidelines for diverticulitis have changed several times in recent years^12,13^ and improving a patient's quality of life is the most critical factor in determining treatment options^14^. With the increase of diverticulitis, non-operative treatment based on dietary restriction and antibiotic therapy has become the mainstay of treatment^15^. More recently, non-operative treatment is preferred even in the case of complicated diverticulitis, such as local intra-abdominal air or abscess formation^5^.

However, if non-operative treatment fails, delayed surgical resection of the large intestine responsible for diverticulitis may be necessary to avoid a severe sepsis process. In these cases, patient management can be much more challenging because of the higher risk of potential sepsis progression and postoperative complications^15^. Therefore, for successful non-operative treatment of diverticulitis, it is essential to predict risk factors for non-operative treatment failure^16^. In addition, there are few studies on the association between the inflammatory index and the prognosis of diverticulitis treatment. This study aims to identify risk factors for the failure of non-operative treatment of acute first-attack colonic diverticulitis, including the inflammatory index.

## Methods

### Patients

**Figure 1:**
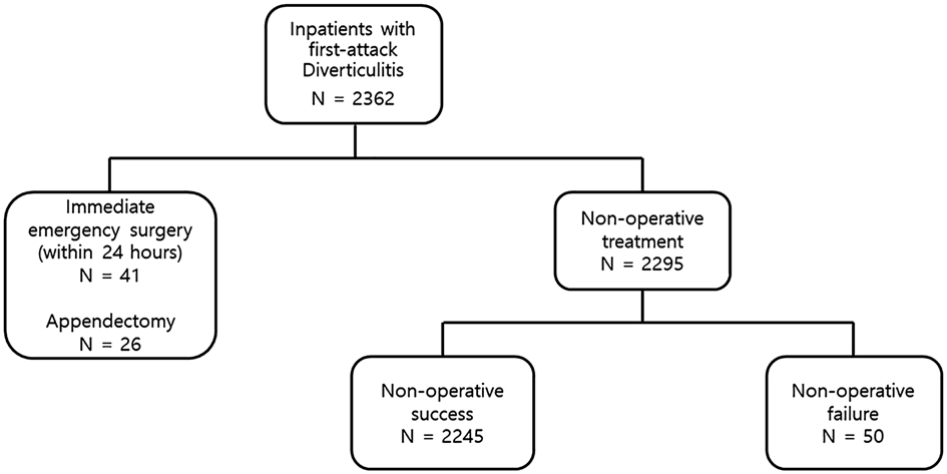
Flow chart of patients group.

We retrospectively reviewed patients diagnosed with colonic diverticulitis from January 2011 to December 2020. This study included patients with only first-attack diverticulitis. Patients were admitted through an outpatient department or emergency department. It was divided into a non-operative treatment success group and a non-operative treatment failure group (Fig. 1). Patients with previous elective or emergency surgery for diverticulitis, history of inflammatory bowel disease, suspected colorectal cancer, appendicitis confirmed by biopsy, and no available follow-up data were excluded. All patients underwent abdominal pelvic computed tomography (CT), and the image of CT was diagnosed by an experienced radiologist. Diverticulitis located in the cecum, ascending colon, and hepatic flexure was classified as right-sided diverticulitis, and diverticulitis located in splenic flexure, descending colon, and sigmoid colon was classified as left-sided diverticulitis. Because the treatment methods for colonic diverticulitis are not different depending on the location, both left and right diverticulitis were included in the study. All diverticulitis patients were classified by the modified Hinchey Grades (mHG). The mHG was classified as follows: grade 0, mild clinical diverticulitis; grade 1a, confined pericolic inflammation or phlegmon; grade 1b, confined pericolic abscess; grade 2, pelvic, intraabdominal, or retroperitoneal abscess; grade 3, generalized purulent peritonitis; and grade 4, fecal peritonitis^17^. Complicated diverticulitis was defined as mHG 2, 3, or 4 grades. This study followed the Declaration of Helsinki and was approved by the Internal Research Board of Chuncheon Sacred Heart Hospital (approval number: 2022-05-021). According to the Clinical Ethics Committee of Hallym University College of Medicine, informed consent was obtained from all subjects and/or their legal guardians.

### Clinical data and inflammatory markers

Clinical data was extracted from electronic patient records at the medical center. Clinical characteristics of patients include age, sex, body mass index (BMI), admission route, hypertension (HTN), diabetes mellitus (DM), cerebrovascular accident (CVA), alcohol history (Alc), smoking history, previous abdominal surgery, location of diverticulitis, modified Hinchey grades (mHGs), and surgical methods. HTN, DM, and CVA were diagnosed and followed up by cardiologists, endocrinologists, and neurologists, respectively. Blood tests for all patients were performed upon admission. Laboratory data of patients such as inflammatory markers were collected and analyzed. White blood cell (WBC) count, neutrophil count, lymphocyte count, monocyte count, platelet count, albumin levels, C-reactive protein (CRP) levels, WBC/lymphocyte ratio (WLR), WBC/neutrophil ratio (WNR), neutrophil/lymphocyte ratio (NLR), lymphocyte/monocyte ratio (LMR), platelet/lymphocyte ratio (PLR), CRP/albumin ratio, and the modified Glasgow prognostic score (mGPS) were included for evaluation. Modified GPS was measured on three scale scores as follows: 0, normal albumin and CRP levels; 1, elevated CRP (> 1.0 mg/dL) levels; and 2, elevated CRP (> 1.0 mg/dL) levels and hypoalbuminemia (< 3.5 g/dL)^18^.

### Non-operative treatment for diverticulitis

Non-operative treatment included no food by mouth, antibiotics, fluid therapy, and pain control regardless of mHG^19^. The initial administered intravenous antibiotics were amoxicillin-clavulanic acid and netilmicinum or 3rd generation cephalosporin and metronidazole depending on renal function. Non-operatively treated patients were discharged from the hospital if they no longer show signs of abdominal pain as measured by the Visual Analogue Scale (VAS) score, the pain is clearly reduced, there is no fever in the previous 48 h, and the inflammatory markers such as WBC or CRP appear normal. The non-operative treatment failure group was defined as a case in which it was judged that surgical resection was necessary because a fever of 37.8 °C or higher persisted or abdominal pain worsened despite non-operative treatment for more than 24 h. In each case, if necessary, two or more colorectal surgeons decided whether to perform surgery due to failure of non-operative treatment.

### Statistical analyses

The primary endpoint was the incidence of surgical resection due to failure of non-operative treatment within 30 days. The groups were compared regarding clinical and inflammatory factors. Differences between categorical variables were compared using the χ^2^ test. The Wilcoxon rank sum test or Student’s *t* test was used for continuous variables with the Shapiro–Wilk normality test. The associations between failure of non-operative management and clinical-inflammatory factors were assessed by using logistic regression analysis. Each factor was assessed in a separate univariate logistic regression analysis. Independent variables reaching a cut-off *p*-value of < 0.2 in univariate analyses were considered for inclusion in a multivariable logistic regression model, and an odds ratio (OR) and 95% CI were calculated for each factor. *P*-value lower than 0.05 was considered statistically significant. The cut-off value for inflammatory index was analyzed using a receiver operating characteristic (ROC) curve. When the area under the curve (AUC) was the largest, the value was fixed as the cut-off value. All statistical analyses were performed using SPSS version 27.0 (IBM Corp., Armonk, NY, USA) after removing patient identifiers from all data sets.

## Results

### Characteristics of patients with colonic diverticulitis

**Figure 2:**
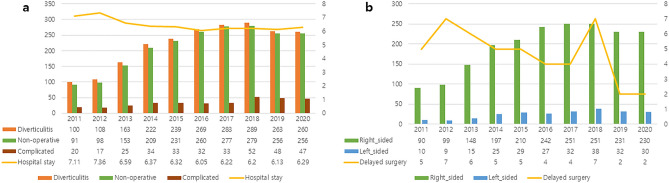
Characteristics of colonic diverticulitis. (**a**) The number of non-operative, complicated, and hospital stays, (**b**) The number of right-sided, left-sided, and delayed surgery.

The overall incidence of colonic diverticulitis increased gradually over the study period and then stabilized in recent years. The annual incidence of diverticulitis inpatients, the number of patients in the non-operative treatment group, and the number of complicated diverticulitis gradually increased. The success rate of annual non-operative treatment gradually increased from 90 to 98%. The hospital stays for diverticulitis showed a gradual decrease (Fig. 2a). Overall, 89.2% (n = 2048) of patients had right-sided diverticulitis and 10.8% (n = 247) had left-sided diverticulitis. The incidence of right-sided diverticulitis was statistically higher than that of left-sided diverticulitis (*p* < 0.005). The annual incidence of right-sided diverticulitis and left-sided diverticulitis increased and then gradually stabilized. The number of surgeries due to failure of non-operative treatment showed a decreasing trend every year (Fig. 2b).

### Characteristics of patients with non-operative treatment

A total of 2295 patients were included, with 1249 (54.4%) men and 1046 women (45.6%). The median age of the total patients was 41 years, ranging from 33 to 91 years. There were 2245 patients in the non-operative success group and 50 patients in the non-operative failure group. The non-operative treatment failure group was older than the success group (*p* = 0.005). In the non-operative success group, 2016 patients had right-sided diverticulitis and 229 patients had left sided diverticulitis. On the other hand, in the non-operative treatment failure group, there were 32 patients with right-sided diverticulitis and 18 patients with left-sided diverticulitis. DM and HTN were more common in the non-operative treatment failure group (*p* = 0.01 and *p* = 0.04, respectively), and the duration of hospitalization was significantly different between two groups (*p* < 0.001). Moreover, complicated diverticulitis was more common in the non-operative treatment failure group (*p* < 0.001). The ratio of sex, initial body temperature, admission route, BMI, CVA, alcohol history, smoking history, and previous abdominal surgery were not different between two groups (Table 1). In patients with successful non-operative treatment, there were no transfers to the intensive care unit, and no patients died. Among the patients with non-operative treatment, 6 patients (0.2%) were hospitalized for more than 30 days, and 49 patients (2%) were readmitted within 30 days after discharge. However, there were no patients who underwent surgical treatment within 1 year after discharge among the patients with non-operative treatment.

**Table 1:** Clinical characteristics.

	Conservative treatment		Total (N = 2295)	*P* value
	Success group	Failure group		
	(N = 2245)	(N = 50)		
Sex				0.511
Female	1026 (45.7%)	20 (40.0%)	1046 (45.6%)	
Male	1219 (54.3%)	30 (60.0%)	1249 (54.4%)	
Age, y	41.0 [33.0;50.0]	46.5 [36.0;61.0]	41.0 [33.0;50.0]	0.005
Hospital stays, d	5.0 [ 5.0; 7.0]	15.0 [12.0;21.0]	5.0 [ 5.0; 7.0]	< 0.001
Admission route				0.290
ER	1291 (57.5%)	33 (66.0%)	1324 (57.7%)	
OPD	954 (42.5%)	17 (34.0%)	971 (42.3%)	
BMI, kg/m^2^	23.6 [21.2;26.2]	23.5 [20.9;26.4]	23.6 [21.2;26.2]	0.887
Hypertension				0.046
No	1913 (85.2%)	37 (74.0%)	1950 (85.0%)	
Yes	332 (14.8%)	13 (26.0%)	345 (15.0%)	
Diabetes mellitus				0.012
No	2161 (96.3%)	44 (88.0%)	2205 (96.1%)	
Yes	84 (3.7%)	6 (12.0%)	90 (3.9%)	
CVA				1.000
No	2236 (99.6%)	50 (100.0%)	2286 (99.6%)	
Yes	9 (0.4%)	0 (0.0%)	9 (0.4%)	
Alcohol history				0.831
No	1200 (53.5%)	28 (56.0%)	1228 (53.5%)	
Yes	1045 (46.5%)	22 (44.0%)	1067 (46.5%)	
Smoking history				0.513
No	1541 (68.6%)	37 (74.0%)	1578 (68.8%)	
Yes	704 (31.4%)	13 (26.0%)	717 (31.2%)	
Previous abdominal surgery				0.271
No	1713 (76.3%)	42 (84.0%)	1755 (76.5%)	
Yes	532 (23.7%)	8 (16.0%)	540 (23.5%)	
Initial body temperature (°C)	36.8 [36.5;37.1]	36.8 [36.5;37.2]	36.8 [36.5;37.1]	0.851
Location of diverticulitis				< 0.001
Right	2023 (90.1%)	32 (64.0%)	2055 (89.5%)	
Left	222 (9.9%)	18 (36.0%)	240 (10.5%)	
mHinchey grade				< 0.001
0	1773 (79.0%)	18 (36.0%)	1791 (78.0%)	
1a	111 (4.9%)	1 (2.0%)	112 (4.9%)	
1b	48 (2.1%)	0 (0.0%)	48 (2.1%)	
2	0 (0.0%)	5 (10.0%)	5 (0.2%)	
3	180 (8.0%)	21 (42.0%)	201 (8.8%)	
4	133 (5.9%)	5 (10.0%)	138 (6.0%)	

Data are n (%) or mean ± SD., Continuous variables expressed as median and interquartile range (IQR 25%–75%).

*ER* emergency room, *OPD* outpatient department, *BMI* body mass index, *CVA* cardiovascular attack.

Regarding the level of inflammatory markers, the lymphocyte count was significantly higher in the non-operative treatment success group (1.8 [1.4; 2.3] × 10^3^/μL) than in the non-operative treatment failure group (1.6 [1.1; 1.8] × 10^3^/μL) (*p* < 0.001). However, there were no differences in WBC counts, neutrophils, and monocytes (*p* = 0.4, *p* = 0.1, and *p* = 0.6, respectively). WNR and LMR were higher in the non-operative treatment success group, but WLR, NLR, and PLR were higher in the non-operative treatment failure group. CRP levels were significantly higher in the non-operative treatment failure group than in the non-operative treatment success group (70.8 mg/dL [26.3; 120.4 mg/dL] vs. 37.2 mg/dL [14.1; 76.6 mg/dL], respectively, *p* = 0.001). On the other hand, albumin levels were significantly higher in the non-operative treatment success group than in the non-operative treatment failure group (4.4 g/dL [4.2; 4.7 g/dL] vs. 4.1 g/dL [3.8; 4.3 g/dL], respectively, *p* < 0.001). Furthermore, the CRP/albumin ratio was significantly higher in the non-operative treatment failure group (*p* < 0.001). Characteristics of inflammatory markers are summarized in Table 2.

**Table 2:** Characteristics of inflammatory markers.

	Conservative treatment		Total (N = 2295)	*P* value
	Success group (N = 2245)	Failure group (N = 50)		
WBC count (× 10^3^/μL)	10.7 [8.4;12.9]	10.9 [9.1;14.3]	10.7 [8.5;12.9]	0.430
Neutrophils (× 10^3^/μL)	7.8 [5.8; 9.9]	8.6 [6.7;10.7]	7.8 [5.8; 9.9]	0.107
Lymphocyte (× 10^3^/μL)	1.8 [1.4; 2.3]	1.6 [1.1; 1.8]	1.8 [1.4; 2.2]	< 0.001
Monocyte (× 10^3^/μL)	0.6 [0.4; 0.8]	0.5 [0.4; 0.8]	0.6 [0.4; 0.8]	0.601
Platelet count (× 10^3^/μL)	240.0 [207.0;283.0]	244.0 [221.0;305.0]	240.0 [207.0;283.0]	0.106
WLR	5.7 [4.3; 7.5]	7.5 [5.3; 9.2]	5.7 [4.3; 7.6]	< 0.001
WNR	1.4 [1.3; 1.5]	1.3 [1.2; 1.4]	1.4 [1.3; 1.5]	0.001
NLR	4.2 [2.9; 5.9]	6.0 [3.7; 7.5]	4.2 [2.9; 6.0]	0.001
LMR	3.1 [2.2; 4.3]	2.5 [1.8; 3.9]	3.1 [2.2; 4.3]	0.017
PLR	132.9 [104.5;171.9]	178.4 [125.0;250.0]	133.3 [104.7;173.2]	< 0.001
CRP (mg/dL)	37.2 [14.1;76.6]	70.8 [26.3;120.4]	37.5 [14.4;77.7]	0.001
Albumin (g/dL)	4.4 [4.2; 4.7]	4.1 [3.8; 4.3]	4.4 [4.2; 4.6]	< 0.001
CRP/Albumin ratio	8.3 [3.2;17.2]	16.8 [6.4;34.0]	8.5 [3.2;17.6]	< 0.001
mGPS				0.001
0	426 (19.0%)	6 (12.0%)	432 (18.8%)	
1	1787 (79.6%)	39 (78.0%)	1826 (79.6%)	
2	32 (1.4%)	5 (10.0%)	37 (1.6%)	

Data are n (%) or mean ± SD., Continuous variables expressed as median and interquartile range (IQR 25%–75%).

*WBC* white blood cell, *WLR* WBC/lymphocyte ratio, *WNR* WBC/neutrophil ratio, *NLR* neutrophil/lymphocyte ratio, *LMR* lymphocyte/monocyte ratio, *PLR* platelet/lymphocyte ratio, *CRP* C-reactive protein, *mGPS* modified Glasgow prognostic scores.

### Risk factors of the failure of non-operative treatment

Logistic regression models were performed to identify risk factors for the failure of non-operative treatment. In the first step, univariable logistic regression was performed for all clinical and inflammatory factors. In univariate logistic regression analysis, it showed that age, HTN, DM, left-sided diverticulitis, mHG, lymphocytes, WLR, NLR, PLR, CRP levels, CRP/albumin ratio, and mGPS were markedly associated with the non-operative treatment failure for diverticulitis.

**Figure 3:**
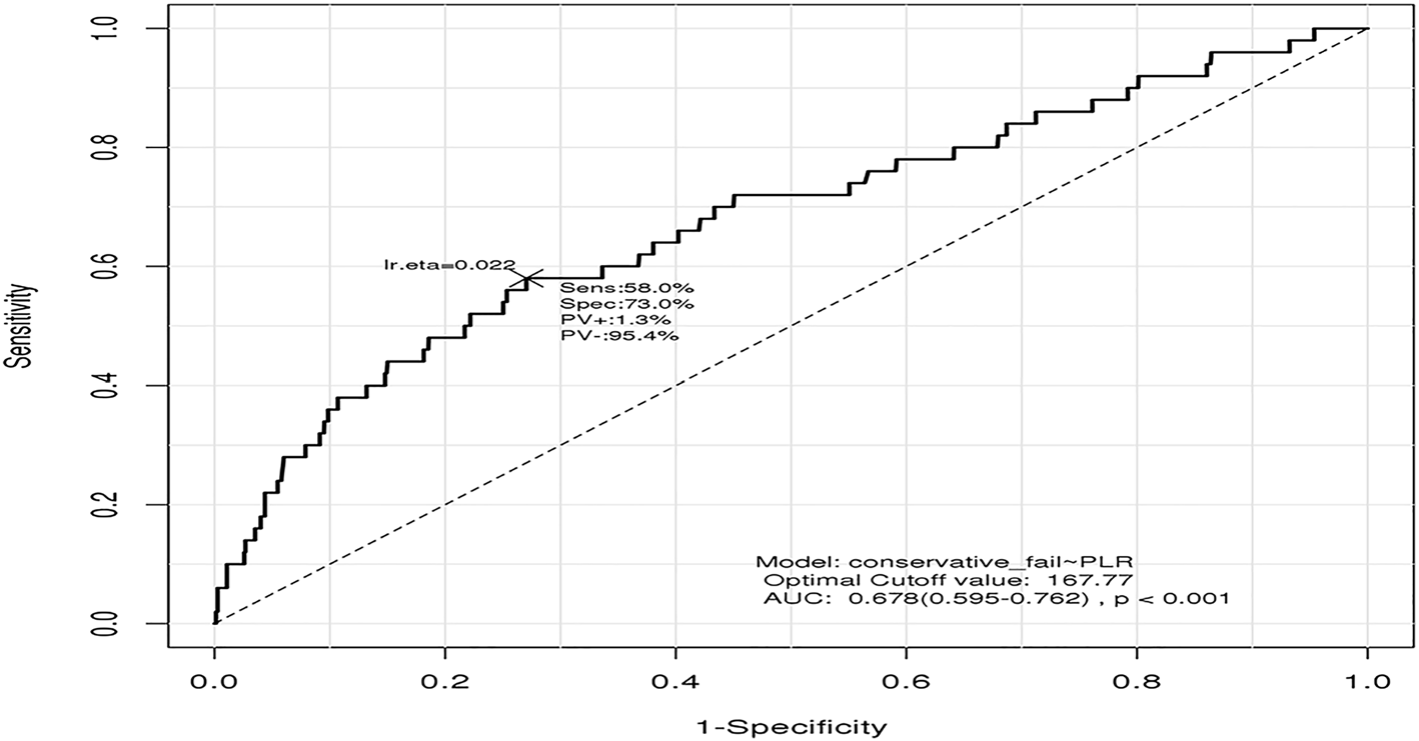
Cut-off value of PLR by ROC curve, *p*: *p* value.

Multivariate analysis was performed with factors related to the non-operative treatment failure in the univariate analysis. DM (OR 2.1, 95% CI: 0.0–0.09, *p* = 0.025) and mHG (OR 6.3, 95% CI: 0.09–0.17, *p* < 0.001) were independent risk factors associated with the non-operative treatment failure. Left-sided diverticulitis was associated with the failure of non-operative treatment than right-sided diverticulitis (OR 4.1, 95% CI: 0.04–0.13, *p* < 0.001). Furthermore, among the inflammatory indices, only PLR was a risk factor associated with the non-operative treatment failure (OR 4.2, 95% CI: 0.05–0.17, *p* < 0.001). Univariate and multivariate analyses are shown in Table 3. In the ROC analysis, the area under the curve (AUC) of PLR was 0.678. An optimized cutoff value of PLR was evaluated as 167.77 based on the ROC analysis with a sensitivity of 58% and a specificity of 73% (Fig. 3).

**Table 3:** Uni- and multivariable logistic regression for conservative treatment failure.

	Univariate analysis				Multivasriate analysis			
	OR	95% CI		*p* value	OR	95% CI		*p* value
Age, years	3.5	0.03	0.11	< 0.001	0.5	− 0.03	0.06	0.588
Sex (male vs. female)	0.8	− 0.05	0.12	0.424				
BMI, kg/m^2^	0.4	− 0.03	0.05	0.687				
Hypertension	2.1	0.01	0.09	0.028				
Diabetes mellitus	2.9	0.02	0.1	0.003	2.2	0.01	0.09	0.025
CVA	− 0.4	− 0.05	0.03	0.654				
Alcoholic history	− 0.3	− 0.05	0.03	0.721				
Smoking history	− 0.8	− 0.06	0.02	0.419				
Left-sided diverticulitis	6.1	0.08	0.17	< 0.001	4.1	0.04	0.13	< 0.001
mHinchey classification	8.1	0.13	0.21	< 0.001	6.2	0.09	0.17	< 0.001
WBC count	0.5	− 0.03	0.05	0.571				
Neutrophil count	1.4	− 0.01	0.07	0.164				
Lymphocyte count	− 3.1	− 0.11	− 0.02	0.002	0.9	− 0.03	0.08	0.333
Monocyte count	− 0.6	− 0.05	0.03	0.55				
WLR	4.5	0.05	0.14	< 0.001	− 0.5	− 1.34	0.78	0.603
WNR	− 1.2	− 0.07	0.01	0.21				
NLR	4.6	0.06	0.14	< 0.001	0.5	− 0.76	1.31	0.606
LMR	− 1.8	− 0.08	0.01	0.74				
PLR	6.3	0.09	0.17	< 0.001	4.2	0.05	0.13	< 0.001
CRP	4.1	0.04	0.13	< 0.001	1.4	0.09	0.21	0.137
CRP/Albumin ratio	4.8	0.06	0.14	< 0.001	1.9	− 0.01	0.24	0.057
mGPS	2.6	0.01	0.1	0.009				

*CI* confidence interval, *OR* odds ratio, *BMI* body mass index, *CVA* cardiovascular attack, *WBC* white blood cell, *WLR* WBC/lymphocyte ratio, *WNR* WBC/neutrophil ratio, *NLR* neutrophil/lymphocyte ratio, *LMR* lymphocyte/monocyte ratio, *PLR* platelet/lymphocyte ratio, *CRP* C-reactive protein, *mGPS* modified Glasgow prognostic scores.

### Surgical treatment of patients with non-operative treatment failure

Despite non-operative treatment, 50 patients underwent delayed surgery for colonic diverticulitis. The surgery was performed in 32 of 2048 patients with right-sided diverticulitis (1.6%) and 18 of 247 patients with left-sided diverticulitis (7.3%). All 32 patients with right-sided diverticulitis underwent right hemicolectomy. Patients with left-sided diverticulitis who underwent surgical resection showed an anterior resection rate of 55.5% (n = 10), a subtotal colectomy rate of 11.1% (n = 2), a Hartmann's operation rate of 22.2% (n = 4), and a left hemicolectomy rate of 11.1% (n = 2). For surgical management, stoma formation occurred most frequently in left-sided diverticulitis with a rate of 22.2% (n = 4) but there was no stoma formation in right-sided diverticulitis (*p* = 0.025). There was no significant difference in the rate of laparoscopic surgery for right-sided (84.4%, n = 27) and left-sided (83.3%, n = 15) diverticulitis (*p* = 0.99). The time difference from hospitalization to surgery due to failure of non-operative treatment was 4.5 [2.5; 7.5] days for right-sided diverticulitis and 4.0 [2.0; 11.0] days for left-sided diverticulitis, and there was no significant difference (*p* = 0.951).

## Discussion

Like reports from several other studies^3,4^, our study showed a gradual increase in diverticulitis over the past decade. The increase in diverticulitis has led to the development of various treatment methods. Although historically surgical treatment for acute complicated diverticulitis has been performed at a much lower threshold, current trends are to prefer non-operative treatment in as many patients as possible to avoid the high mortality associated with surgery^20^. Furthermore, conservative management including non-operative therapy for advanced acute diverticulitis has a success rate of up to 95%^20^. The development of antibiotics and the change in the treatment paradigm to non-operative treatment have improved the success rate of non-operative treatment. Non-operative treatment for diverticulitis has been continuously developed, supported by a shorter hospital stay and a reduction in surgery. Our study showed a reduction in surgically managed patients from 6.5 to 0.7% due to non-operative treatment. During the same period, the length of hospitalization for non-operative treatment decreased from 7.3 to 6.2 days. Other previous studies also have shown similar results^21,22^. However, despite non-operative treatment according to guidelines, 2.2% of patients in our study (right diverticulitis: 1.5%, left diverticulitis: 7.3%) underwent delayed surgery within 30 days. Non-operative treatment failure rates ranged from 0 to 14.3%, and for right diverticulitis, the pooled rate was 2.5%^23^. In a previous meta-analysis, the treatment failure rate was 6–7% for left-sided diverticulitis^24^. There is an urgent need to allow individualized treatment decisions about whether to manage early surgically or non-operatively in the acute episode of diverticulitis. Therefore, predicting risk factors for non-operative treatment failure is important for treatment strategy.

The most common used staging system for colonic diverticulitis is the Hinchey classification, which has been gradually modified since its development in 1978. Indications for immediate emergency surgery for the treatment of diverticulitis can be confirmed with CT images with mHG, but imaging has limitations in predicting failure of non-operative treatment. Therefore, other factors, such as inflammatory indices, may be meaningful to predict the failure of non-operative treatment. Inflammatory indices, including PLR determined based on the naïve complete blood cell counts, are known to be better predictors of prognosis in patients diagnosed with cancer or other acute diseases^25,26^. However, the effect of inflammatory indices on the non-operative treatment prognosis of diverticulitis has not yet been established. Tests for inflammatory markers to predict the severity of inflammation are simple to measure, as the ratio can be easily determined from differential whole blood cell counts, and the cost of the test is inexpensive.

One study showed symptoms resulting from diverticulitis appear to have a more organic cause from a diseased segment of the colon rather than a functional bowel disorder^27^. Therefore, these patients may have persistent inflammation, which require surgical treatment. Despite various non-operative therapies, patients with persistent inflammation represent a population with a high probability of potential septic progression that requires surgery for acute diverticulitis. This supports that the inflammatory index indicative of persistent inflammation can help predict surgical treatment due to non-operative treatment failure. Another interesting possibility may be to investigate the nutritional status of patients with diverticulitis. In some studies, a decrease in albumin in patients with the diverticular disease has been shown to increase the length of hospital stay^28^. However, in our study, nutritional markers such as albumin were not a significant factor for non-operative treatment failure. We investigated various markers such as NLR, LMR, PLR, CRP/albumin ratio, and mGPS to evaluate risk factors for the failure of non-operative treatment of colonic diverticulitis.

The lymphocyte is an essential component of the white blood cells which play a vital role in the regulation of the inflammatory microenvironment and can be reflected in peripheral blood measurable parameters. Platelets are important mediators of clotting, thrombosis, and inflammation because they secrete several molecules involved in inflammation^29^. In addition, platelets, which identify inflammatory processes, are characterized by regulating other types of cells such as neutrophils, promoting adhesion to lymphocytes^30,31^. Measuring the divergence among these WBC components has been believed to be more accurate at predicting poor clinical outcomes than measuring each one individually^30^. However, a few studies have reported a prognostic role of PLR in diverticulitis. Mari et al. reported higher mean PLR values in patients who were operated compared to those treated with non-operative treatment for only complicated diverticulitis^32^. Zager et al.^33^ suggested that PLR may be related to an inflammatory burden, which may explain the value of PLR in predicting the interval of time between episodes of diverticulitis. Our study demonstrated that PLR was a risk factor for non-operative treatment failure regardless of complicated or uncomplicated diverticulitis. Additionally, the cutoff value of PLR derived from the ROC curve showed high sensitivity and specificity.

The rate of complicated diverticulitis at the time of the first attack episode ranged from 6.2 to 12.4%^34,35^. In our study, the uncomplicated case was present in 85% and the complicated case in 15% of patients among the first-attack diverticulitis. Multivariate analysis for complicated diverticulitis showed that mHG (OR 3.9, 95% CI: 0.1–0.31, *p* < 0.001) and PLR (OR 2.2, 95% CI: 0.02–0.3, *p* = 0.027) were significant risk factors of surgery after non-operative treatment failure (Supplemental Table 1). Since complicated diverticulitis is an advanced state of inflammation, there is a limit to predicting non-operative treatment failure with mHG alone. Therefore, it is significant that PLR, an inflammatory index, was identified as a risk factor for the non-operative treatment failure of complicated diverticulitis. On the other hand, in uncomplicated diverticulitis, left-sided diverticulitis (OR 2.1, 95% CI: 0.01–0.09, *p* = 0.035) and PLR (OR 2.1, 95% CI: 0.01–0.09, *p* = 0.036) were found to be significant risk factors for non-operative treatment failure (Supplemental Table 2). Anatomically, the right colon is located primarily in the retroperitoneum, which can limit the spread of inflammation, and the sigmoid colon, the most common site of left diverticulitis, is located in the abdominal cavity, making it easier to spread inflammation^36^. In addition, since it is difficult to determine the exact degree of inflammation in uncomplicated diverticulitis with imaging tests and simple inflammation markers, this result may suggest that PLR reflects the degree of inflammation for treatment prognosis. Therefore, our study demonstrated that PLR, which reflects the degree of inflammation, is a significant risk factor for the failure of non-operative treatment, regardless of uncomplicated or complicated diverticulitis.

There are several limitations to this study. First, there are limitations of retrospective studies and the difference in sample size between groups. Second, the time from symptom onset to hospital arrival was not evaluated in this study, and it is possible that this time interval influenced the outcome of inflammatory markers. However, this study analyzed large data for total colonic diverticulitis regardless of the location of diverticulitis, uncomplicated or complicated diverticulitis. It is a meaningful study to identify risk factors for non-operative treatment failure in the era when non-operative treatment is mainstream. In the future, long-term, large-scale, well-designed randomized controlled trials are needed in the setting of patients with the adjustment for detailed relevant clinical and inflammatory factors.

## Conclusions

Platelet to lymphocyte ratio is a potential risk factor for the non-operative treatment failure of acute first-attack colonic diverticulitis. These findings may improve decision-making for subsequent surgery. Therefore, patients with higher PLR during non-operative treatment should be monitored with special caution.

## Supplementary Information

Supplementary Table 1.

Supplementary Table 2.

## Data Availability

Data are available from the authors upon reasonable request and with permission.
