# Supplementary material for: Platelet to lymphocyte ratio is a risk factor for failure of non-operative treatment of colonic diverticulitis
Source: Sci Rep. 2023 Mar 16;13:4377. doi: 10.1038/s41598-023-31570-3 (PMC10020164; doi:10.1038/s41598-023-31570-3)
Supplement: Supplementary file 1 — Supplementary Table 1. [file 41598_2023_31570_MOESM1_ESM.docx]

|  | **Univariate analysis** | | | | **Multivasriate analysis** | | | |
| --- | --- | --- | --- | --- | --- | --- | --- | --- |
|  | **OR** | **95% CI^a^** | | ***p* value** | **OR** | **95% CI** | | ***p* value** |
| Age, years | 1.8 | -0.01 | 0.2 | 0.068 | 0.9 | 0.07 | 0.18 | 0.366 |
| Sex (male vs. female) | 0.6 | -0.15 | 0.28 | 0.543 |  |  |  |  |
| BMI, kg/m^2^ | 0.3 | -0.09 | 0.12 | 0.732 |  |  |  |  |
| Hypertension | 1.8 | -0.01 | 0.2 | 0.076 | 0.2 | -0.13 | 0.11 | 0.84 |
| Diabetes mellitus | 3.2 | 0.06 | 0.27 | 0.002 | 2.7 | 0.04 | 0.24 | 0.008 |
| CVA | -0.5 | -0.13 | 0.08 | 0.656 |  |  |  |  |
| Alcoholic history | 0.3 | -0.09 | 0.12 | 0.774 |  |  |  |  |
| Smoking history | -0.4 | -0.13 | 0.08 | 0.636 |  |  |  |  |
| Left-sided diverticulitis | 3.1 | 0.06 | 0.27 | 0.002 | 1.4 | -0.03 | 0.2 | 0.163 |
| mHinchey classification | 4.4 | 0.13 | 0.34 | <0.001 | 3.9 | 0.1 | 0.31 | <0.001 |
| WBC count | -0.6 | -0.14 | 0.07 | 0.54 |  |  |  |  |
| Neutrophil count | -0.1 | -0.12 | 0.1 | 0.870 |  |  |  |  |
| Lymphocyte count | -1.7 | -0.2 | 0.01 | 0.074 | -0.1 | -0.15 | 0.14 | 0.99 |
| Monocyte count | -1.1 | -0.17 | 0.04 | 0.244 |  |  |  |  |
| WLR | 2.1 | 0.01 | 0.22 | 0.038 | 1.8 | 0.12 | 5.04 | 0.061 |
| WNR | -0.1 | -0.11 | 0.1 | 0.953 |  |  |  |  |
| NLR | 2.2 | 0.01 | 0.22 | 0.032 | 1.8 | -0.18 | 4.97 | 0.068 |
| LMR | -0.2 | -0.12 | 0.1 | 0.845 |  |  |  |  |
| PLR | 3.6 | 0.09 | 0.3 | <0.001 | 2.2 | 0.02 | 0.3 | 0.027 |
| CRP | 1.7 | -0.01 | 0.2 | 0.083 | 1.8 | 0.04 | 1.07 | 0.071 |
| CRP/Albumin ratio | 2.3 | 0.02 | 0.23 | 0.02 | 1.9 | -0.08 | 1.15 | 0.089 |
| mGPS | 1.6 | -0.02 | 0.19 | 0.118 |  |  |  |  |

**Supplement Table 1.** logistic regression for conservative treatment failure for complicated diverticulitis. OR = odds ratio, CI = confidence interval, BMI = body mass index, CVA = cardiovascular attack, WBC = white blood cell, WLR = WBC/lymphocyte ratio, WNR = WBC/neutrophil ratio, NLR = neutrophil/lymphocyte ratio, LMR = lymphocyte/monocyte ratio, PLR = platelet/lymphocyte ratio, CRP = C-reactive protein, mGPS = modified Glasgow prognostic scores.
