# Supplementary material for: Platelet to lymphocyte ratio is a risk factor for failure of non-operative treatment of colonic diverticulitis
Source: Sci Rep. 2023 Mar 16;13:4377. doi: 10.1038/s41598-023-31570-3 (PMC10020164; doi:10.1038/s41598-023-31570-3)
Supplement: Supplementary file 2 — Supplementary Table 2. [file 41598_2023_31570_MOESM2_ESM.docx]

|  | **Univariate analysis** | | | | **Multivasriate analysis** | | | |
| --- | --- | --- | --- | --- | --- | --- | --- | --- |
|  | **OR** | **95% CI** | | ***p* value** | **OR** | **95% CI** | | ***p* value** |
| Age, years | 1.6 | -0.01 | 0.08 | 0.095 | 0.2 | -0.04 | 0.05 | 0.846 |
| Sex (male vs. female) | 0.3 | -0.07 | 0.1 | 0.741 |  |  |  |  |
| BMI, kg/m^2^ | -0.1 | -0.05 | 0.04 | 0.85 |  |  |  |  |
| Hypertension | 0.2 | -0.04 | 0.05 | 0.837 |  |  |  |  |
| Diabetes mellitus | -0.8 | -0.06 | 0.03 | 0.405 |  |  |  |  |
| CVA | -0.3 | -0.05 | 0.04 | 0.793 |  |  |  |  |
| Alcoholic history | -0.8 | -0.06 | 0.03 | 0.392 |  |  |  |  |
| Smoking history | -0.9 | -0.07 | 0.02 | 0.348 |  |  |  |  |
| Left-sided diverticulitis | 2.8 | 0.02 | 0.11 | 0.006 | 2.1 | 0.01 | 0.09 | 0.035 |
| mHinchey classification | -0.6 | -0.06 | 0.03 | 0.534 |  |  |  |  |
| WBC count | 0.1 | -0.04 | 0.05 | 0.948 |  |  |  |  |
| Neutrophil count | 0.4 | -0.04 | 0.05 | 0.697 |  |  |  |  |
| Lymphocyte count | -1.5 | -0.08 | 0.01 | 0.14 | -0.3 | -0.08 | 0.06 | 0.782 |
| Monocyte count | -0.8 | -0.06 | 0.02 | 0.379 |  |  |  |  |
| WLR | 1.3 | -0.02 | 0.02 | 0.216 |  |  |  |  |
| WNR | -0.6 | -0.06 | 0.03 | 0.536 |  |  |  |  |
| NLR | 1.2 | -0.02 | 0.07 | 0.246 |  |  |  |  |
| LMR | -0.2 | -0.05 | 0.04 | 0.825 |  |  |  |  |
| PLR | 2.6 | 0.01 | 0.11 | 0.011 | 2.1 | 0.01 | 0.09 | 0.036 |
| CRP | 0.8 | -0.02 | 0.06 | 0.386 |  |  |  |  |
| Albumin | -3.9 | -0.13 | -0.04 | <0.001 | -3.4 | -0.12 | -0.03 | <0.001 |
| CRP/Albumin ratio | 1.1 | -0.02 | 0.07 | 0.251 |  |  |  |  |
| mGPS | 1.3 | -0.01 | 0.08 | 0.172 | 0.7 | -0.03 | 0.03 | 0.439 |

**Supplement Table 2.** logistic regression for conservative treatment failure for uncomplicated diverticulitis. OR = odds ratio, CI = confidence interval, BMI = body mass index, CVA = cardiovascular attack, WBC = white blood cell, WLR = WBC/lymphocyte ratio, WNR = WBC/neutrophil ratio, NLR = neutrophil/lymphocyte ratio, LMR = lymphocyte/monocyte ratio, PLR = platelet/lymphocyte ratio, CRP = C-reactive protein, mGPS = modified Glasgow prognostic scores.
